# Role of Reputation in Sustainable Performance of Online Crowdsourcing Vendors: An Explanation From Transaction Cost Theory

**DOI:** 10.3389/fpsyg.2021.756134

**Published:** 2021-11-23

**Authors:** Xiao Han, Mengxiao Xue, Wenhui Song

**Affiliations:** School of Business, Qingdao University, Qingdao, China

**Keywords:** crowdsourcing, reputation, vendors, sustainable performance, online labor market, transaction cost theory (TCT), panel data

## Abstract

This study reveals a new finding on the impact of reputation growth on crowdsourcing vendors’ sustainable performance in different modes of markets using fixed-effect panel data regression models. To this end, we extract data from a large Chinese crowdsourcing platform named zbj.com for the period of 2012–2014, which was a key stage for the establishment of market diversification. Based on different transaction modes, the study divides the markets on the crowdsourcing platform into task-based market (TBM) and employment market (EPM). By applying the multiple framework, the empirical results exhibit a negative and significant effect of vendors’ reputation on participation rate (PR) in TBM and EPM. At the same time, reputation also has a consistent effect on vendors’ revenue share (RS) of each market. Moreover, this study shows that the significant reputation impact on PR and RS of EPM will be, respectively, weakened and strengthened in fixed-price mode and customized mode when vendors participate more in large-scale projects. The findings suggest that the growth of reputation will promote market transfer of vendors, that is, showing different sustainability in different markets, which will lead to uneven development of the crowdsourcing markets. By adopting the perspective of transaction cost theory (TCT), this study elaborates and analyses these phenomena and derives corresponding policy implications.

## Introduction

In recent years, the rise of online crowdsourcing has come to provide a broader platform for enterprises. This new outsourcing model, born in the internet era, has become increasingly known and accepted (Chandler and Kapelner, 2013). Although the current online labor market is still developing, crowdsourcing platforms already have considerable numbers of registered users (Chen and Horton, 2016). Vendors seek new ways to participate in employment because they cannot find suitable traditional paid jobs; consequently, many are turning to online labor markets such as crowdsourcing platforms (Afuah and Tucci, 2012). This outsourcing model can make full use of the advantages of internet technology, accelerate the globalization of labor, and significantly improve the efficiency of human resource allocation. Crowdsourcing platforms such as Upwork.com and Freelancer.com enable vendors to complete various simple or complex projects for employers worldwide. With the development of crowdsourcing platforms, the market types of crowdsourcing have become increasingly diversified. Vendors can choose markets with different trading modes to obtain higher efficiency and benefits (Estellés-Arolas and González-Ladrón-de-Guevara, 2012).

In a broad sense, crowdsourcing tasks can be divided into microtasks, design-creative tasks, and macrotasks (Cheng et al., 2015). Microtasks are a simple and straightforward form of crowdsourcing. Every microtask can be completed simply and cheaply by the public; however, with the accumulation of tasks, a large project can be achieved (Majima et al., 2017). Successful examples include OpenStreetMap and crowdsourcing logistics. Design-creative tasks are another type of crowdsourcing (Blazquez-Resino et al., 2020; Al-Kumaim et al., 2021). With the advent of the Internet, some public-oriented design competitions can be implemented through crowdsourcing, which enables the demand-side companies to break the enterprise’s geographical or internal boundaries and obtain the most valuable works (Bayus, 2013; Buenadicha-Mateos et al., 2019). Macrotasks involve enterprises hiring vendors on crowdsourcing platforms to deliver satisfactory solutions that cannot be generated internally (Clemons, 2009; Satzger et al., 2013; Liu et al., 2014). Such tasks usually require more upfront investment and devotion of considerable resources and energy: examples include business planning, software development, and engineering design. This article focuses on design-creative tasks and macrotasks, which are generally arranged through a third-party online crowdsourcing platform.

Existing studies classify market types on crowdsourcing platforms mainly from the perspectives of task features. Howe (2006), for instance, differentiates between three types of markets, i.e., crowdsourcing idea game, crowdsourced problem solving, and prediction markets. Afuah and Tucci (2012) distinguish crowdsourcing markets between tournament-based and collaboration-based crowdsourcing. Taeihagh (2017) generalizes three types of crowdsourcing on microtasks in virtual labor markets, tournament crowdsourcing and open collaboration. These studies pay less attention to the classification from transaction modes. Through the investigation of the existing mainstream platforms, we have introduced a new classification focusing on design-creative tasks and macrotasks based on transaction modes. According to the latest Alexa Traffic Rank, we selected some high-traffic crowdsourcing websites and analyzed their market types (see Table 1). Therefore, this study divides the crowdsourcing market into task-based markets (TBM) and employment markets (EPM). TBM is the initial popular market of crowdsourcing platforms. After the employer publishes a task, vendors in the market compete to be selected to undertake it, and the employer chooses satisfactory works (reward mode) or qualified vendors (bidding mode) according to their preferences. EPM is a later form of market that emerges after a crowdsourcing platform develops to a particular stage. On some crowdsourcing platforms, vendors can set up their stores and package service products (fixed-price mode) or wait for employers to hire (customized mode). Projects with the fixed-price mode are better suited to small-scale projects, whereas the customized mode is more suitable for large-scale projects.

**TABLE 1:** Market types of crowdsourcing platforms.

	Task-based market		Employment market	
	Reward mode	Bidding mode	Fixed-price mode	Customized mode
Freelancer.com		•	•	•
Upwork.com		•		•
Guru.com		•	•	•
Peopleperhour.com		•	•	•
Kaggle.com	•			
Zbj.com	•	•	•	•

*Note: The black circle means this mode is applied by the platform.*

There have been various studies on the influencing factors of crowdsourcing vendors’ working behavior. First, many scholars have analyzed vendors’ online performance. Demographics including age, gender, education, and number of income sources can explain the patterns in workers’ participation and how workers engage in different types of work (Chen et al., 2019). Subtask heterogeneity also plays a significant role on the formation of the online performance in crowdsourcing labor markets (Mourelatos et al., 2020a). Monitoring with economic penalties may activates social norms for honesty and promotes honest reporting in an online labor market (Reffett et al., 2019). The factors that affect the pricing behavior of crowdsourcing vendors mainly include product costs, competitive dynamics, and virtual costs (Hong et al., 2016). Another study has indicated that, like offline labor markets, monopsony also be present in online crowdsourcing labor markets (Dube et al., 2020). Second, crowdsourcing quality issue is a common concern of related literature. One study found that the quality of crowdsourcing microtasks completed by vendors is not mainly affected by monetary incentives (Zheng et al., 2011). At the same time, the quality of complex crowdsourcing tasks is relatively high when the degree of cooperation is high or low, but a medium degree of cooperation is associated with low quality (Bullinger et al., 2010). Moreover, complex crowdsourcing projects with higher prices usually attract the participation of more experienced vendors and obtain higher quality results (Liu et al., 2014).

As in most online markets, reputation play a critical role in affecting crowdsourcing vendors’ behavior (Kokkodis and Ipeirotis, 2016). Employers mainly pay attention to reputation when choosing vendors and use it as a quality signal (Bolton et al., 2004). This is why researchers have increasingly come to focus on how to measure reputation in crowdsourcing markets. There are three main reputation mechanisms: (a) establish a feedback rating system (Yoganarasimhan, 2013); (b) review qualifications, for instance through online verification of work experience (Agrawal and Tambe, 2016), a gold membership system (Banker and Hwang, 2008), and third-party certification (Goes et al., 2010); and (c) effective two-party interaction (Gefen and Carmel, 2008). Research to date has explored various reputation mechanisms to solve the problems of information asymmetry in online labor markets, and provided foundations for the development of online transactions. However, the complexity of online interactions has so far limited understanding of the impact of crowdsourcing vendors’ reputation. A few studies have done some related research. Evidence have been found that the earnings a contractor obtains from working through crowdsourcing market positively correlates with higher reputation scores (Gandini et al., 2016). In more detail, reputation has a significant impact on the transaction type of fixed-price contracts, but no obvious impact for time-material contracts (Lin et al., 2018). Moreover, raters were candid when feedback was private, but when feedback suddenly became public, reputations began inflating (Filippas et al., 2017).

A review of the literature shows that little research (Ma et al., 2020) has been involved into the reputation impact on sustainable performance of crowdsourcing vendors in different markets. Moreover, while scholars have conducted a series of studies of transaction costs in electronic transactions (Hill, 1990; Teo and Yu, 2005; Thomassen et al., 2016), few have addressed the field of crowdsourcing. This article, therefore, draws on transaction cost theory to verify how reputation affects sustainable performance of vendors for different market modes in a crowdsourcing platform. By empirically analyzing crowdsourcing vendors’ performance in various markets, this article aims to provide both theoretical and empirical basis for crowdsourcing vendors and platforms to formulate better strategies.

## Theoretical Background and Hypotheses

Transaction cost theory (TCT) was proposed originally by Coase (1937) to explain the existence of firms, and was further developed by Williamson (1981, 1985). The adoption of transaction costs in online transactions research also has a long history (Teo and Yu, 2005; Wu et al., 2014; Liang et al., 2021; Mohammad et al., 2021). TCT explains why a transaction subject chooses a particular form of transaction instead of others. Different transaction costs in various labor markets can lead to multiple segmentations (Masters and Miles, 2002). On the other words, given that all other factors are equal, a customer will go with a channel that has lower transaction costs whether online or offline (Liang and Huang, 1998).

Transaction cost theory has two behavioral assumptions, bounded rationality and opportunism, among them, uncertainty is an important reason (Nooteboom, 1992). A specific analytical perspective demonstrates that, in the TBM, it is difficult for vendors to identify trusted partners (Bapna et al., 2001; Farrell et al., 2017; Reffett et al., 2019), which will increase uncertainties of the transaction and further increase transaction costs (Teo and Yu, 2005; Anwar et al., 2006; De Schepper et al., 2015). On the other hand, in the EMP, customization may diminish uncertainties, which in turn will lead to lower transaction costs (Mohammad et al., 2021). This may indicate that transaction costs in TBM are higher than in EMP. This study further analyses and verifies this prediction below.

Transaction costs involved in online transactions can be classified as searching costs, monitoring costs, and adapting costs (Teo and Yu, 2005). The searching cost is that associated with the time or effort expended by vendors in finding proper tasks and selecting employers. Monitoring costs are costs spent by vendors to ensure that contracts are faithfully executed. Adapting cost is the cost vendors bear to deal with exceptions during contract implementation or costs associated with after-sales services (Liang et al., 2021). Based on this classification, we conducted an online survey of crowdsourcing vendors to confirm the difference in transaction costs between different market modes. The questionnaire was tested with 312 vendors enrolled in both TBM and EPM of a large Chinese crowdsourcing platform named zbj.com. The design of the questionnaire items refers to the relevant literature (Teo and Yu, 2005; Liang et al., 2021), as shown in Appendix 1. Table 2 summarizes the mean value of transaction costs perceived by vendors in various crowdsourcing markets. The data show that, in general, TBM has higher transaction costs than EPM, which is coincident with the previous forecast.

**TABLE 2:** Mean value of transaction cost in different markets of survey data.

		Searching cost	Monitoring cost	Adapting cost
TBM	Reward mode	4.653	4.313	2.032
	Bidding mode	4.327	4.565	3.587
EPM	Fixed-price mode	2.575	2.128	2.563
	Customized mode	2.839	3.221	3.295

For further analysis, we also found evidence consistent with other studies. First, in TBM, since vendors need to filter large number of tasks fitting for them, the searching costs are relatively high. By contrast, in EPM, more accurate information can be easily obtained, which dramatically reduces vendors’ searching costs in transactions (Anderson and Gatignon, 1986; Leiblein, 2003). Second, there is more obvious information asymmetry and uncertainty in TBM, so the supplier has less control over the transaction process, which leads to an increase in monitoring costs (De Schepper et al., 2015; Choi and Contractor, 2016). Moreover, in the fixed-priced mode of EPM, most of the tasks have relatively fixed solutions, meaning that vendors’ monitoring costs are minimal. Third, as adapting costs involving more ex post costs, the difference in average adapting costs between the two markets is not obvious. Thus, for vendors, the overall transaction costs in EPM are much lower than those in TBM. When the perceived transaction costs are relatively high, vendors’ willingness to continue participating will be relatively low; conversely, when the perceived transaction costs are relatively low, vendors’ willingness to continue participating in that market will be stronger (Liang and Huang, 1998). Accordingly, when conditions permit, vendors may tend to choose the EMP for conducting transactions (Yasuda, 2005; Reuer and Ragozzino, 2014).

Through a review of the literature, reputation is a common indicator that affects online user choices and transaction costs (De Schepper et al., 2015; Thomassen et al., 2016; Lin et al., 2018). In crowdsourcing platform-based transactions, the lack of face-to-face communication means that reputation has a significant impact on crowdsourcing vendors (Zhou et al., 2008; Kokkodis and Ipeirotis, 2016; Lin et al., 2018). Therefore, based on the perspective of TCT, this study chooses reputation as a main influencing factor to measure vendors’ market participation and revenue sources. In this scenario, after first entering a crowdsourcing market, a vendor with little reputation has to generate a higher reputation score by participating in TBM, with high transaction costs. Once the vendors acquire enough reputation, the lower transaction costs will likely motivate vendors to set up their stores and attract more employers in EPM. As the vendor’s reputation grows, it will become more feasible for them to sustainably participate in EPM. On this basis, we propose the following hypothesis:

**H1**: As the reputation of crowdsourcing platform vendors improves, the proportion participating in the TBM will decrease and the proportion participating in the EPM will increase.

H1 aims to verify the influence of reputation on the sustainable performance of the vendors’ crowdsourcing market. On this basis, this research will further examine how the growth of the vendors’ reputation affects their revenue. The previous setting suggests that as the reputation of vendors improves, their income will increasingly come from EPM. The following hypothesis is therefore proposed:

**H2**: As the reputation of crowdsourcing platform vendors improves, their revenue share in TBM will decrease and their revenue share in the EPM will increase.

To describe in more detail the impact of reputation growth on the vendors’ sustainable performance, we will consider scales of the tasks refer to other related studies (Barthélemy and Quélin, 2006; Mohammad et al., 2021). The link between task scales and transaction costs is quite straightforward. As the scale increases, the number of contingencies in the contract will goes up, it thus becomes more expensive to write, monitor and enforce (Balakrishnan and Wernerfelt, 1986). Transaction costs will be high in fulfilling more contractual requirements when vendors participating in large-scale projects (Williamson, 1991; Bartheìlemy, 2001).

From the overall crowdsourcing market, the reward mode of TBM accounts for a relatively small proportion. This study therefore does not distinguish between the reward and bidding modes of TBM in the empirical part. Thus, we analyze three markets: TBM (Market 1), fixed-price mode EPM (Market 2), and customized mode EPM (Market 3). According to our investigation, Market 2 is better suited to small-scale tasks and Market 3 is more suited to large-scale tasks. The relatively higher transaction costs of large-scale projects mean that when vendors move from TBM to EPM, the scale of the task will moderate the impact of reputation. Specifically, an increase in vendors’ participation in large-scale projects will negatively moderate their participation and revenue share in Market 2 but positively moderate their participation and revenue share in the Market 3. The following assumptions are therefore made:

**H3a**: When vendors participate in more large-scale crowdsourcing projects, the influence of their reputation on their willingness to participate in the fixed-price mode EPM (Market 2), and on their revenue share in this market, will weaken.

**H3b**: When vendors participate in more large-scale crowdsourcing projects, the influence of their reputation on their willingness to participate in the customized mode EPM (Market 3), and on their revenue share in this market, will increase.

**FIGURE 1:**
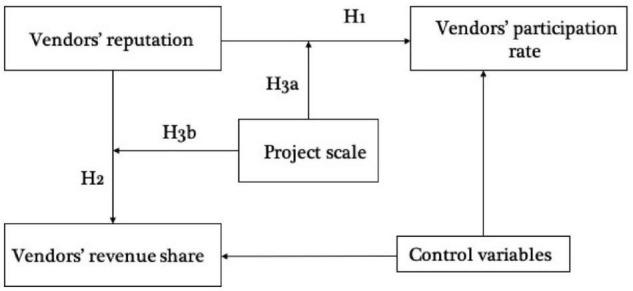
Research framework.

The research framework is shown in Figure 1.

## Materials and Methods

### Sample Selection

This article conducts empirical research on crowdsourcing platform vendors. The specific data source is Zbj.com, one of the largest comprehensive crowdsourcing platforms in China. Zbj.com was founded in 2006 and was initially mainly based on the bidding/reward mode (TBM). In 2012 the platform introduced the store mode (EPM) to offer more benefits for vendors and increase the efficiency of its crowdsourcing markets. Interviews with two managers from Zbj.com in 2019 highlighted that 2012–2014 was a critical period for EPM, as it progressed from inception to prosperity. Given that many vendors entered EPM at this stage, the influence of reputation and task characteristics on vendors’ sustainable performance in different markets is easy to observe.

In seeking to study the vendors’ performance in different markets, we collected all transaction data of the top 1,000 active vendors during the 2-year transition period from September 2012 to August 2014. After excluding vendors who participated in very many or very few projects, along with those who undertook invalid transactions, we finally selected more than 500,000 valid tasks in which 589 vendors participated in TBM and EPM over the 2 years. This served as the research sample for our empirical analysis.

The selected sample vendors are all small teams with 3–10 employees who have registered for 1–3 years on the platform by the year 2012. The vendors distributed in various regions of China, among which Chongqing (where the platform is located) and the eastern coastal areas are the main ones. The industries involved by the sample vendors include logo design, development and IT, writing and translating, marketing strategy and Video editing.

### Measures

To analyze the extent to which reputation influences the sustainable performance of vendors in different crowdsourcing markets, we adopt panel data regression models. This section focuses on the research design used in the study. After first setting out the definition and measurement approach for each variable, we then present the research models.

#### Sustainable Performance of Different Crowdsourcing Markets

To accurately reflect the sustainable performance of vendors in different markets, this article selects the vendors’ market participation rate (*PTC*_*m,it*_) and market revenue share (*RVN*_*m,it*_) as the dependent variables, where *m* represents a different crowdsourcing market, and *t* represents a period (each month is one time period, and there are a total of 24 time periods). The two variables, respectively, represent (a): the participation rate of vendor *i* in crowdsourcing market *m* in period *t*, and (b): the revenue share of vendor *i* in crowdsourcing market *m* in period *t*.

#### Reputation Value

This article draws on previous research (Yoganarasimhan, 2013; Kokkodis and Ipeirotis, 2016; Lin et al., 2018) by selecting the vendor’s reputation value as the independent variable (*RPT*_*t*_), measured by the average reputation value of vendor *i* in period *t*. As vendors continue to accumulate completed projects in the crowdsourcing market, their reputation value will continue to increase. Reputation value is calculated following the rules set by the platform^1^.

#### Control Variables

The following control variables are included:

• Participation rate of a vendor in large projects (*LAP*_*t*–1_).

The transaction costs of vendors in the bidding mode is relatively high, so participating in large projects in the task-based market will significantly increase transaction costs. The participation rate in large projects will, therefore, affect the vendor’s market participation and revenue. This variable is expressed as the proportion of large-scale projects in which vendor *i* participates during period *t*−1.

• Vendor’s project experience (*EXP*_*t*–1_).

In crowdsourcing markets, experience is an essential determinant of the likelihood of being hired (Gefen and Carmel, 2008; Ma et al., 2020). The more projects in which a vendor participates, the greater their project experience and the better their ability to judge the pros and cons of various market choices, which will affect their market participation choices. This variable is expressed as the cumulative total number of projects in which vendor *i* participated during period *t*−1.

• Vendor’s winning-bid rate (*SBW*_*t*–1_/*LBW*_*t*–1_).

Most new vendors in a task-based market initially have a very low winning rate for their bids. However, with the accumulation of project experience and reputation, vendors’ bid-winning rate will gradually increase. The bid-winning rate inevitably impacts on vendors’ willingness to participate in the market. Of them, large-scale projects and small-scale projects somewhat differ in nature because small-scale projects are always repetitive low-tech items. Therefore, this study takes the cumulative winning rate for large-scale projects and for small-scale projects as two independent control variables to construct the model. The two variables are, respectively, expressed as the bid-winning rate for large-scale and small-scale projects of vendor *i* in period *t*−1.

• Project’s overall market share (*MKS*_*mt*_).

The overall market share of projects represents the relative number of projects in a certain period of the market, which is bound to impact each vendor’s market selection at that time. This variable is expressed as the proportion of all projects in market *m* in time period *t*: *m* = 1 represents Market 1; *m* = 2 represents Market 2; and *m* = 3 represents Market 3.

• Average price difference between markets (Δ*MKP*_21,_*_*t*_*/Δ*MKP*_31,_*_*t*_*).

If the overall average price of items in a crowdsourcing market is higher, then the willingness of vendors to participate in the market will increase. We therefore select two control variables: the average price difference between Market 2 and Market 1 in period *t*, and the average price difference between Market 3 and Market 1 in period *t*.

Table 3 details the descriptive statistics. Since not all the vendors started to participate in the tasks at the beginning of the selected period, some of the observations of 589 vendors in 24 periods are missing. Thus, the observations numbers of variables related to vendors (*RPT*_*t*_,*LAP_*t*_*_–1_, *EXP*_*t*–1_, *SBW*_*t*–1_, *LBW*_*t*–1_) are 12,278. The remaining variables are not associated with vendors, and only related to the market in the certain period. The observation numbers are 14,136, among them, the value of each vendor in the same period is equal. Moreover, because the reputation value (*RPT*_*t*_) and vendors’ experience (*EXP*_*t*–1_) are much larger than other variables, the natural logarithm is taken in the model to get higher precision. In addition, since there exists negative value (minimum value = −1379) for *MKS*_21,t_, the natural logarithm is taken after normalization in the data processing.

**TABLE 3:** Descriptive statistics.

Variable	Observation	Mean	SD	Min	Max
*RPT*_*t*_	12,278	28,875	12,389	0	1,966,783
*LAP*_*t*–1_	12,278	0.596	0.268	0	1
*EXP*_*t*–1_	12,278	65.761	48.577	0	1,896
*SBW*_*t*–1_	12,278	0.356	0.128	0	1
*LBW*_*t*–1_	12,278	0.327	0.176	0	1
*MKS*_1*t*_	24	0.622	0.066	0.488	0.781
*MKS*_2*t*_	24	0.104	0.076	0	0.215
*MKS*_3*t*_	24	0.275	0.060	0.168	0.436
Δ*MKP*_21,_*_*t*_*	24	3,711	1,976	−1,379	26,762
Δ*MKP*_31,_*_*t*_*	24	2,440	908	482	4,084

*See section “Appendix 2” for variable details.*

### Empirical Models

To test the theoretical framework mentioned above, we establish the following panel data models:

**Model 1:**


(1) $$\begin{array}{c} \;PTC_{m,it}=\beta_{0}+\beta_{1}lnRPT_{i,t}+\beta_{2}LAP_{i,t-1}+\beta_{3}lnRPT_{i,t}\times \\ LAP_{i,t-1}+\beta_{4}lnEXP_{i,t-1}+\beta_{5}SBW_{i,t-1}+\beta_{6}LBW_{i,t-1}+\\ \beta_{7}MKS_{mt}+\beta_{8}ln\text{Δ}MKP_{21,t}+\beta_{9}ln\text{Δ}MKP_{31,t}+\mu_{it}+v_{i} \end{array}$$

**Model 2:**


(2) $$\begin{array}{c} \;RVN_{m,it}=\beta_{0}+\beta_{1}lnRPT_{i,t}+\beta_{2}LAP_{i,t-1}+\beta_{3}lnRPT_{i,t}\times \\ LAP_{i,t-1}+\beta_{4}lnEXP_{i,t-1}+\beta_{5}SBW_{i,t-1}+\beta_{6}LBW_{i,t-1}+\\ \beta_{7}MKS_{mt}+\beta_{8}ln\text{Δ}MKP_{21,t}+\beta_{9}ln\text{Δ}MKP_{31,t}+\mu_{it}+v_{i} \end{array}$$

where *m* = 1, 2, 3, respectively, represents Market 1, Market 2, and Market 3; *i* (*i* = 1,…,589) and *t* (*t* = 1,…,24), respectively, represent the *i*_*th*_ vendor and *t*_*th*_ time period.

## Results

### Sample Description

**FIGURE 2:**
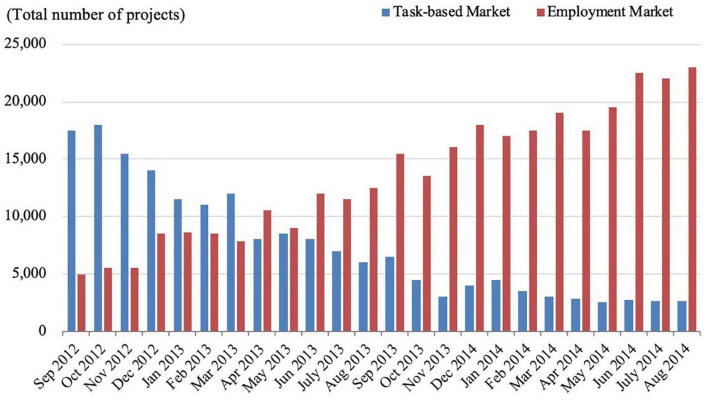
Distribution of the total number of projects in which sample vendors participated.

**FIGURE 3:**
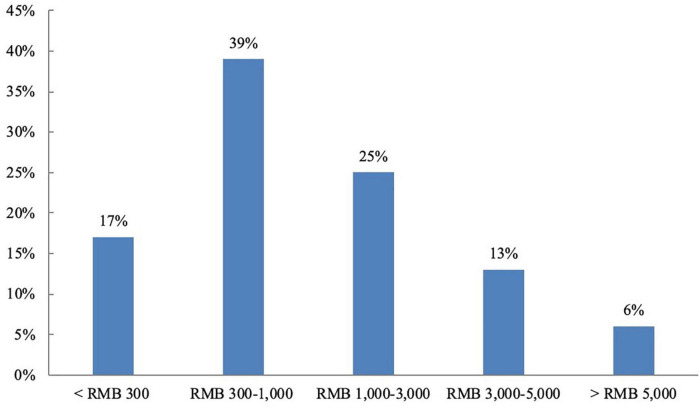
Proportion of sample vendors participating in projects of different scales.

**FIGURE 4:**
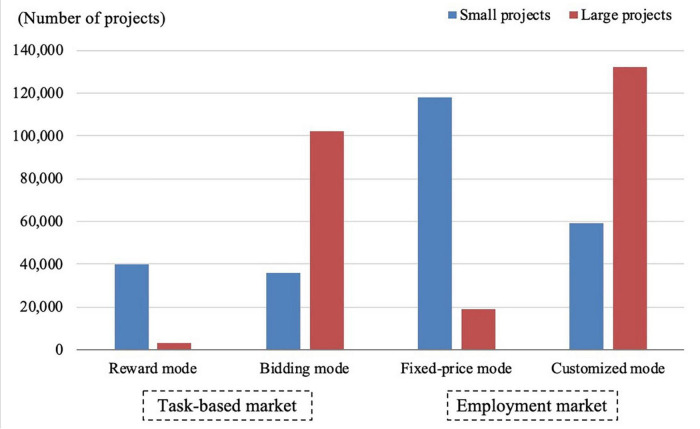
Comparison of project types in which sample vendors participated.

Based on the sample data, we divide the study period into 24 calendar months to construct a time series and add the vendor profiles to form panel data. Regarding the overall distribution of projects in the sample (Figure 2), we see a significant shift over time in the proportion of projects in each market, with the proportion of projects in the EPM gradually increasing. Figure 3 depicts the distribution of projects of different scales based on project value. Projects valued at RMB 300–1,000 account for the most transactions (39%), followed by projects valued at RMB 1,000–3,000 (25%), below RMB 300 (17%), RMB 3,000–5,000 (13%), and above RMB 5,000 (6%). We also sought to verify whether task characteristics influence vendors’ market selection by making a simple distinction between large- and small-scale projects. Because the sample mean is very sensitive to outliers, the median RMB 805 is adopted for this purpose. On this basis, we counted the total number of large- and small-scale projects in which the sample vendors participated in the crowdsourcing markets (see Figure 4). The results show that in TBM, the reward mode is suitable for small projects while the bidding mode is more suitable for large projects. In EPM, the fixed-price mode is more often used for relatively simple and highly reproducible small projects, while the customized mode is generally used for large-scale projects.

**FIGURE 5:**
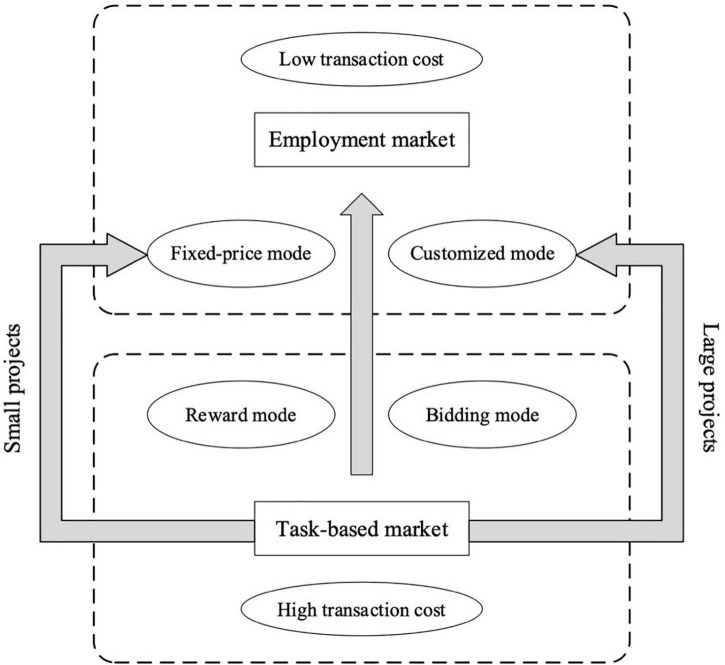
Market transition trends of crowdsourcing vendors.

Based on the above analysis, Figure 5 depicts the market transition trend presented by the sample. It shows that projects generally shift from TBM with higher transaction costs to EPM with lower transaction costs. At the same time, small projects flow more toward the fixed-price mode, whereas large projects flow more toward the customized mode. The following empirical analysis will further verify the influence of vendors’ reputation growth on the sustainable performance in difference markets.

### Empirical Results

Tables 4, 5 report the results from the fixed-effect regression models, respectively, analyzing the influence of the vendor’s reputation and other variables on the vendor’s market participation (Model 1) and revenue share (Model 2).

**TABLE 4:** Panel fixed-effects regression analysis results of vendors’ market participation rate.

Variable	Task-based market (TBM) (Market 1)	Employment market (EPM)	
		Fixed-price mode (Market 2)	Customized mode (Market 3)
	
	Model 1	Model 2	Model 3
*RPT*_*t*_	−0.211*** (0.032)	0.059** (0.017)	0.143** (0.031)
*LAP*_*t*–1_	−0.304* (0.025)	0.365*** (0.012)	0.297*** (0.025)
*RPT*_*it*_ × *LAP*_*t*–1_	−0.099** (0.052)	−0.199*** (0.029)	0.081** (0.045)
*EXP*_*t*_	1.233 (0.028)	−0.031* (0.012)	−0.261 (0.019)
*SBW*_*t*–1_	−0.053* (0.028)	0.160*** (0.013)	0.046* (0.025)
*LBW*_*t*–1_	0.117*** (0.022)	−0.004 (0.010)	0.046* (0.021)
*MKS*_*m* *t*_	0.356*** (0.060)	0.254** (0.038)	0.631*** (0.131)
Δ*MKP*_21,_*_*t*_*	0.482 (0.106)	0.139** (0.048)	−0.313** (0.089)
Δ*MKP*_31,_*_*t*_*	−0.687 (0.275)	−0.059 (0.186)	1.232*** (0.365)
Constant	−0.129 (0.021)	−0.033 (0.009)	−1.299 (0.128)
Observations	12,278	12,278	12,278
*R* ^2^	0.5362	0.6733	0.6289

(1) Standard errors in parentheses, (2) ***p < 0.01, **p < 0.05, *p < 0.1.

**TABLE 5:** Panel fixed-effects regression analysis results of vendors’ market revenue share.

Variable	Task-based market (TBM) (Market 1)	Employment market (EPM)	
		Fixed-price market (Market 2)	Customized market(Market 3)
	
	Model 4	Model 5	Model 6
*RPT*_*t*_	−0.221*** (0.015)	0.085*** (0.033)	0.199*** (0.005)
*LAP*_*t*–1_	−0.214*** (0.036)	0.449** (0.022)	0.427*** (0.017)
*RPT*_*it*_ × *LAP*_*t*–1_	−0.266*** (0.027)	−0.263*** (0.035)	0.054** (0.018)
*EXP*_*t*_	0.738* (0.123)	0.014 (0.016)	−0.054* (0.193)
*SBW*_*t*–1_	0.053** (0.015)	0.185*** (0.026)	0.045 (0.039)
*LBW*_*t*–1_	0.400*** (0.066)	−0.013 (0.125)	−0.006 (0.010)
*MKS*_*m* *t*_	0.343 (0.127)	0.331** (0.062)	0.740*** (0.115)
Δ*MKP*_21,_*_*t*_*	0.252 (0.021)	0.181** (0.037)	−0.253*(0.092)
Δ*MKP*_31,_*_*t*_*	−1.136** (0.322)	−0.040 (0.028)	1.319** (0.369)
Constant	0.858 (0.258)	−0.114 (0.035)	−1.762 (0.056)
Observations	12,278	12,278	12,278
*R* ^2^	0.5189	0.7527	0.7066

(1) Standard errors in parentheses, (2) ***p < 0.01, **p < 0.05, *p < 0.1.

#### Reputation Effects on Vendors’ Market Participation Rate

In Table 4, the coefficients of vendor’s reputation (*RPT*_*t*_) in the three models are, respectively, significantly negative (*r* = −0.211, *p* < 0.01), significantly positive (*r* = 0.059, *p* < 0.05), and significantly positive (*r* = 0.143, *p* < 0.05). These results indicate that an increase in reputation lowers a vendor’s participation in TBM but increases the participation in EPM. In other words, as vendors’ reputation improves, the willingness of participation in TBM (Market 1) will gradually decrease and their willingness to participate in EPM (Market 2 and Market 3) will gradually increase. This is consistent with H1.

#### Reputation Effects on Vendors’ Market Revenue Share

In Table 5, the coefficients on vendor’s reputation (*RPT*_*t*_) in the three models are, respectively, significantly negative (*r* = −0.221, *p* < 0.01), significantly positive (*r* = 0.085, *p* < 0.01), and significantly positive (*r* = 0.199, *p* < 0.01). These results indicate that as vendors’ reputation improve, their revenue share in EPM (Market 2 and Market 3) will increase. This is consistent with H2.

#### The Moderating Effect of Project Scale

In terms of the control variables, the vendor’s participation rate in large-scale projects (*LAP*_*t*–1_) has significantly positive coefficients for Markets 2 (*r* = 0.365, *p* < 0.01; *r* = 0.449, *p* < 0.05) and Market 3 (*r* = 297, *p* < 0.01; *r* = 0.427, *p* < 0.01) in Tables 4, 5. These results indicate that when vendors participate in more large-scale projects, both their participation and the share of their revenue generated in EMP will increase. These findings are consistent with our prior expectations. The moderating effect of task scale is tested by introducing the interaction “*RPT*_*t*_ × *LAP*_*t*–1_” into the model. The results in Tables 4, 5 show that, in both models, the coefficient of this interaction is significantly negative in Market 2 (*r* = −0.199, *p* < 0.01; *r* = −0.263, *p* < 0.01), and the coefficient of Market 3 is significantly positive (*r* = 0.081, *p* < 0.05; *r* = 0.054, *p* < 0.05). These results show that the reputation impact on vendors’ participating in customized mode EPM (Market 3) will increase as the participation rate of large-scale projects increases, and the reputation influence of vendors’ participating in the fixed-price mode EPM (Market 2) will be weakened when enrolled into more large-scale projects. Similarly, the vendor’s reputation effect on revenue share in fixed-price mode EPM (Market 2) and customized mode EPM (Market 3) will be relatively weakened and enhanced with a rise of the large-scale projects participation rate. These findings are consistent with H3a and H3b.

### Robustness Checks

Robustness checks were conducted to further verify the findings. As mentioned above, the sample vendors serve different industries. Considering the influence of task characteristic, we grouped vendors who enrolled in different types of tasks. Based on the classification of crowdsourcing tasks (Cheng et al., 2015), we distinguish between design-creative tasks and macrotasks for different industries. Development and IT tasks, writing and translating tasks and video editing tasks can be considered as macrotasks. On the other hand, Logo design tasks and marketing strategy tasks are generally design-creative tasks. From this perspective, we divide crowdsourcing vendors into creative groups and non-creative groups, containing 272 and 317 vendors, respectively. Regressions are conducted for evaluating the reputation impact on participation rate in different markets of both groups.

Model 7, Model 9, and Model 11 in Table 6 shows the empirical results of creative groups while Model 8, Model 10, and Model 12 provides the result of non-creative group. Model 7 and Model 8 demonstrates that reputation negatively moderates the vendors’ participation rate of TBM in both scenario (*r* = −0.109, *P* < 0.05; *r* = −0.423, *P* < 0.01). The coefficients of *RPT*_*t*_ in Model 9–Model 12 are significantly positive, indicating vendors from both groups have a strong willingness to participate in EPM when reputation increase continuously. In addition, both coefficient value and significance level of creative group are higher than non-creative ones. This indicates that the results are more prominent in non-creative group. For the interaction item “*RPT*_*t*_ × *LAP*_*t*–1_,” we also got the same conclusion as the original models. To summarize, based on the analysis of two vendor groups in different industries, we can assume that the task characteristics will not alter the reputation impact on vendors’ performance in different crowdsourcing markets. This shows that the empirical results are robust to a certain extent.

**TABLE 6:** Robustness checks.

Variable	Task-based market (TBM) (Market 1)		Employment market (EPM)			
			Fixed-price mode (Market 2)		Customized mode (Market 3)	
	
	Model 7	Model 8	Model 9	Model 10	Model 11	Model 12
*RPT*_*t*_	−0.109** (0.016)	−0.423*** (0.067)	0.037** (0.012)	0.046*** (0.011)	0.233** (0.027)	0.187*** (0.039)
*LAP*_*t*–1_	−0.289*** (0.024)	−0.321*** (0.053)	0.159** (0.008)	0.287*** (0.015)	0.129*** (0.016)	0.235*** (0.007)
*RPT*_*it*_ × *LAP*_*t*–1_	−0.095** (0.034)	−0.178*** (0.041)	−0.161** (0.017)	−0.225*** (0.024)	0.077** (0.052)	0.065*** (0.030)
*EXP*_*t*_	0.738* (0.123)	−0.188 (0.027)	−0.033* (0.029)	−0.027** (0.011)	−0.173 (0.021)	−0.248 (0.034)
*SBW*_*t*–1_	−0.085** (0.039)	−0.036** (0.014)	0.213*** (0.006)	0.145*** (0.018)	0.056* (0.043)	0.029 (0.013)
*LBW*_*t*–1_	0.103** (0.071)	0.299*** (0.085)	−0.012 (0.023)	−0.008 (0.030)	0.075* (0.032)	0.151* (0.077)
*MKS*_*m* *t*_	0.470** (0.093)	0.508** (0.045)	0.210** (0.028)	0.332*** (0.016)	0.418*** (0.092)	0.586*** (0.101)
Δ*MKP*_21,_*_*t*_*	0.176 (0.031)	0.297 (0.026)	0.197** (0.056)	0.134* (0.034)	−0.222** (0.049)	−0.403** (0.061)
Δ*MKP*_31,_*_*t*_*	−0.987 (0.276)	−1.413* (0.309)	−0.038 (0.191)	−0.055 (0.267)	0.957*** (0.348)	1.525*** (0.266)
Constant	−0.234 (0.046)	−0.118 (0.025)	−0.081 (0.015)	−0.024 (0.007)	−0.763 (0.055)	−1.628 (0.181)
Observations	5,933	6,345	5,933	6,345	5,933	6,345
*R* ^2^	0.5067	0.6503	0.6161	0.7046	0.6755	0.7221

(1) Standard errors in parentheses, (2) ***p < 0.01, **p < 0.05, *p < 0.1.

### Discussion

Overall, the results indicate that vendors on the crowdsourcing platform have gradually shifted from the TBM to EPM in line with improvement in their reputation over time. Vendors’ sustainable performance in EPM is strong, and the revenue share generated in EPM has increased accordingly. This is because transaction costs are higher for crowdsourcing vendors in TBM. Once vendors’ have a sufficiently established reputation to access opportunities in the employment market, they will become more willing to divert their attention from, and perhaps even quit, the TBM.

For EPM, our analysis revealed the customized mode primarily includes large and high-value projects, while the fixed-price market is for packaged products with relatively low-value projects. When the vendor participates in more large-scale projects, the influence of their reputation on the participation rate and revenue share will be weakened in the fixed-price EPM but relatively more robust in the customized EPM.

## Conclusion and Limitations

This article drew on transaction cost theory to explore the relationship between the growth of vendors’ reputation, the scale of crowdsourcing tasks, and the vendors’ sustainable performance in different types of crowdsourcing markets. Building on previous studies (Liu et al., 2014; Hong et al., 2016; Lin et al., 2018; Mourelatos et al., 2020b), this research provides new evidence of online crowdsourcing vendors’ behavior from a unique perspective, and enriches related research in the field of crowdsourcing. This research also expands the application of TCT to the field of social business in the new era.

This study sourced data from a large Chinese crowdsourcing platform and established a panel fixed-effect model. The results indicate that crowdsourcing vendors tend to shift from TBM to EPM as their reputation improves, although this depends on the market environment and their capabilities. It should be noted that vendors have to establish a sufficiently strong reputation before entering EPM. Once their reputation reaches a higher level, vendors will become less willing to continue participating in TBM and more inclined to participate in EPM. Vendors may use TBM to improve their reputation and prepare themselves for entering EPM. However, within EPM, projects in the fixed-price mode are relatively more straightforward, repeatable, and decomposable than those in the customized mode, which is relatively more suitable for large-scale projects. The results also demonstrate that when vendors accept more large-scale projects, reputation plays a minor role in the participation rate and revenue share of the fixed-price mode EPM and has a more significant impact on the customized mode EPM.

The conclusion of this study provides a theoretical logic to explain the mechanism of TCT in the crowdsourcing markets. Extend studies apply TCT to examine the market migration due to different transaction costs, and verify that users tend to choose channels with lower transaction costs (Liang and Huang, 1998; Teo and Yu, 2005; Wu et al., 2014; Mohammad et al., 2021). Considering the situation of crowdsourcing, based on a vendors’ survey and literature review, this study introduces a new category of crowdsourcing markets and analyses the level of transaction costs in different markets, verifying that vendors also prefer channels with lower transaction costs in crowdsourcing environment. On the other hand, the premise that transaction costs can work is that other conditions of users are equal (Liang and Huang, 1998). The differences in other factors may cause the effect of transaction costs to be unobservable. Therefore, at the same time, we introduce reputation as an important measure of vendor condition to verify whether it is possible to enter channels with lower transaction costs as vendor conditions change. This study explain that the improvement of reputation can increase vendors’ participation rate and revenue share of the market with lower transaction costs, which provides new empirical evidence for the role of reputation in vendors’ market sustainable performance under TCT theory. In addition, the positive moderation effect of large-scale participation rate on reputation also shows that reputation plays a more significant role in market migration when the difference in transaction costs is greater.

This research yields several managerial implications for the operators of crowdsourcing platforms. Just like the traditional labor market, there appear to be large resource differences among crowdsourcing markets, which lead to significant preferences of crowdsourcing vendors. Vendors who enter a premium market and gain a strong reputation early may then behave monopolistically (Dube et al., 2020), making the more profitable EPM saturated and difficult to enter. In view of this, crowdsourcing platforms should further regulate market order, continuously explore new business models, improve reputation mechanisms, and avoid imbalances in income distribution and brain drain. Platforms should aim to assist vendors of different ability levels and with different field specialisms, thereby building more appropriate market segments and better promoting the coordinated development of various markets. Moreover, platforms should assume more social responsibilities and better maintain the rights of crowdsourcing vendors. For example, individuals and SMEs that are new to the market should be given more support.

This study has several shortcomings that need to be addressed by future research. First, the analyzed sample includes observations from only one crowdsourcing labor platform. Although the chosen platform is one of the largest crowdsourcing platforms in China, with many active projects and vendors, it conceivably has unique characteristics that may prevent generalizing the research findings to other online labor markets. Second, the sample also lacks data on some vendor characteristics, including the age, region, and gender of the vendor team, none of which were controlled for in the study. Although such information is often not observable when trading in a crowdsourcing labor market, these individual characteristics may need to be considered in this type of researches. Further studies could focus on expanding the sample to include various global crowdsourcing platforms. In addition, a diversified methodology like natural experiment could be applied to undertake an in-depth assessment of the research context.

## Data Availability Statement

The raw data supporting the conclusions of this article will be made available by the authors, without undue reservation.

## Author Contributions

XH has contributed to the hypotheses, empirical models, data collection, data analysis, article writing, proofreading, and final approval. MX has contributed to data analysis, validation of results, article writing, and revision. WS have contributed to data analysis, drawing limitations, future directions, and the conclusion of the study. All authors contributed to the article and approved the submitted version.

## Conflict of Interest

The authors declare that the research was conducted in the absence of any commercial or financial relationships that could be construed as a potential conflict of interest.

## Publisher’s Note

All claims expressed in this article are solely those of the authors and do not necessarily represent those of their affiliated organizations, or those of the publisher, the editors and the reviewers. Any product that may be evaluated in this article, or claim that may be made by its manufacturer, is not guaranteed or endorsed by the publisher.

## Footnotes

1 The reputation value calculation rules provided by the platform. Basic rules: reputation growth value = transaction amount × growth coefficient Growth coefficient rules: (i) After the transaction is completed, employers will make evaluation. Positive comment: growth coefficient = 1; Neutral comment: growth coefficient = 0.5; Negative Comment: growth coefficient = 0, no growth value will be accumulated. (ii) Employers actively make positive comment: the growth coefficient +0.1; Employers actively make positive comment and the number of words is greater than 15 words, the growth coefficient +0.2. (iii) Repeat customer transactions: if the vendor conducts two or more transactions with employers, after the second and subsequent transactions are completed and positive comment are made, the growth coefficient +0.5.

## Appendix

**APPENDIX 1:** Questionnaire items (Transaction costs).

Transaction costs	Items
**Searching cost**	I spend a lot of time and effort looking for information in the mode of market
	I wish I had more time to look for information in the mode of market
**Monitoring cost**	I can easily monitor the whole transaction process in the mode of market
	I spent a lot of time and effort monitoring whether the tasks I participated are work well in the mode of market
	The mode of market provides me the flexibility to solve any problems with the tasks
**Adapting cost**	The mode of market allows me easily adjust any requirement of the tasks when necessary
	The mode of market allows me easily deal with unexpected changes

**APPENDIX 2:** Detail explanation of control variables.

Control variables	Description
*LAP*_*t*–1_	Proportion of large-scale projects in which vendor *i* participated in period *t*−1
*EXP*_*t*–1_	Cumulative total number of projects in which vendor *i* participated in period *t*−1
*SBW*_*t*–1_	Winning-bid rate for small-scale projects of vendor *i* in period *t*−1
*LBW*_*t*–1_	Winning-bid rate for large-scale projects of vendor *i* in period *t*−1
*MKS*_*m* *t*_	Proportion of all projects in market *m* in period *t* in the markets
Δ*MKP*_21,_*_*t*_*	Average price difference between Market 2 and Market 1 in period *t*
Δ*MKP*_31,_*_*t*_*	Average price difference between Market 3 and Market 1 in period *t*
